# Provider-Initiated Late Preterm Births in Brazil: Differences between Public and Private Health Services

**DOI:** 10.1371/journal.pone.0155511

**Published:** 2016-05-19

**Authors:** Maria do Carmo Leal, Ana Paula Esteves-Pereira, Marcos Nakamura-Pereira, Jacqueline Alves Torres, Rosa Maria Soares Madeira Domingues, Marcos Augusto Bastos Dias, Maria Elizabeth Moreira, Mariza Theme-Filha, Silvana Granado Nogueira da Gama

**Affiliations:** 1 Department of Epidemiology and Quantitative Methods in Health, Sérgio Arouca National School of Public Health, Oswaldo Cruz Foundation, Rio de Janeiro, Brazil; 2 National Institute of Women, Children and Adolescents Health Fernandes Figueira, Oswaldo Cruz Foundation, Rio de Janeiro, Brazil; 3 National Health Agency, Ministry of Health, Rio de Janeiro, Brazil; 4 National Institute of Infectious Diseases, Oswaldo Cruz Foundation, Rio de Janeiro, Brazil; PreTel Inc, UNITED STATES

## Abstract

**Background:**

A large proportion of the rise in prematurity worldwide is owing to late preterm births, which may be due to the expansion of obstetric interventions, especially pre-labour caesarean section. Late preterm births pose similar risks to overall prematurity, making this trend a concern. In this study, we describe factors associated with provider-initiated late preterm birth and verify differences in provider-initiated late preterm birth rates between public and private health services according to obstetric risk.

**Methods:**

This is a sub-analysis of a national population-based survey of postpartum women entitled “Birth in Brazil”, performed between 2011 and 2012. We included 23,472 singleton live births. We performed non-conditional multiple logistic regressions assessing associated factors and analysing differences between public and private health services.

**Results:**

Provider-initiated births accounted for 38% of late preterm births; 32% in public health services and 61% in private health services. They were associated with previous preterm birth(s) and maternal pathologies for women receiving both public and private services and with maternal age ≥35 years for women receiving public services. Women receiving private health services had higher rates of provider-initiated late preterm birth (rate of 4.8%) when compared to the ones receiving public services (rate of 2.4%), regardless of obstetric risk–adjusted OR of 2.3 (CI 1.5–3.6) for women of low obstetric risk and adjusted OR of 1.6 (CI 1.1–2.3) for women of high obstetric risk.

**Conclusion:**

The high rates of provider-initiated late preterm birth suggests a considerable potential for reduction, as such prematurity can be avoided, especially in women of low obstetric risk. To promote healthy births, we advise introducing policies with incentives for the adoption of new models of birth care.

## Introduction

Numbers of preterm births have recently risen in middle and high-income countries [1]. This leads to increased costs for the healthcare system, as well as potential harm to newborns and their families [2, 3]. In 2005, a committee of experts organized by the National Institute of Child Health and Human Development of the National Institutes of Health (NIH) in the USA suggested the term “late preterm” should be used for newborns with GA between 34 0/7 and 36 6/7 weeks [3]. The rise of preterm births worldwide is largely due to late preterm [4], which is partially related to increased obstetric interventions designed to reduce maternal and fetal complications, such as labour induction and pre-labour caesarean section (C-section) [5]. Nevertheless, there is often not enough evidence to recommend these interventions in many cases where they have been performed [6]; i.e., the medical records fail to show appropriate medical indications, suggesting they are not indicated. Brazil is known for having one of the world’s highest rates of C-section [7]. In 2013, levels reached 56%; 43% among women receiving public healthcare and 88% among women receiving private healthcare at childbirth [8]. Many C-sections are reported as conducted for non-medical reasons [9], including those among preterm births (S1 Table) [10].

The impact of late prematurity on newborn health was long neglected as the length and weight of these infants are similar to full-term infants, and they appear not to require much extra healthcare at birth [11]. However, the last weeks before completing 40 weeks gestation are a critical period for brain and lung development [4]. Therefore, being born even slightly before reaching full-term (39–40 weeks gestation) may have significant implications for newborn health [12]. The first studies to suggest that late prematurity poses similar risks to overall prematurity (although at lower frequency and severity) were published a decade ago [3, 13]. Compared with those born at full-term, late preterm infants are more likely to experience respiratory distress syndrome, intraventricular haemorrhage, neonatal or infant death, and long-term neurodevelopmental problems [14–16].

According to the WHO, Brazil is among the 10 countries that contribute most to the burden of prematurity worldwide [17]. However, the magnitude of late prematurity in the country was unknown until 2012, because official health statistics reported gestational ages (GA) at broad intervals [18]. The first national survey into labour and birth was performed in Brazil in 2011 [19, 20]. This described the prevalence of prematurity, its subtypes and associated factors. The study found a prematurity rate of 11.5%, among which 74% were late-premature and 39% were provider-initiated (either determined by labour induction or pre-labour C-section) (S2 Table) [21].

Public and private health services in Brazil employ the same birth care professionals, but organise obstetric care differently [22]. In public health services, medical practitioners work in shifts and pregnant women receive prenatal care in Health Units with obstetric physicians or nurses. Women receive birth care by the staff on duty in the hospital to which they are referred. In private health services, the same physician who provides prenatal care usually provides birth care in a hospital with an open clinic staff; a factor associated with its higher C-section rates [22]. It is unknown, however, whether these idiosyncrasies of public and private obstetric care in Brazil lead to differences in the rates of provider-initiated late preterm birth.

Therefore, this study aims to verify differences in the rates of provider-initiated late preterm birth between public and private health services, in women of low and high obstetric risk. We also describe the subtypes and factors associated with provider-initiated late preterm birth in both sources of healthcare provision.

## Materials and Methods

### Data source

The “Birth in Brazil study” was a national population-based study of postpartum women and their newborns, conducted from February 2011 to October 2012. It recruited a complex sample of 266 hospitals, with 90 postpartum women interviewed in each hospital and a total 23,894 women. Further information about the sample is detailed elsewhere [23]. In the sampled hospitals, women were invited to participate if they gave birth to a live newborn (regardless of weight or GA) or to a stillborn (with birth weight ≥500g and/or GA ≥22 weeks) during the observation period.

Face-to-face interviews were held with the postpartum women during their hospital stay. Further data about the women and their newborns were collected from their medical records and extracted from photographs of prenatal care cards. Women and newborns remaining as inpatients, including those transferred to other hospitals, were tracked for up to 28 days and 42 days, respectively. Further details on data collection have been published elsewhere [19].

### Participants

For the current analysis, we excluded stillbirths, women with unknown GA and multiple pregnancies.

**Fig 1:**
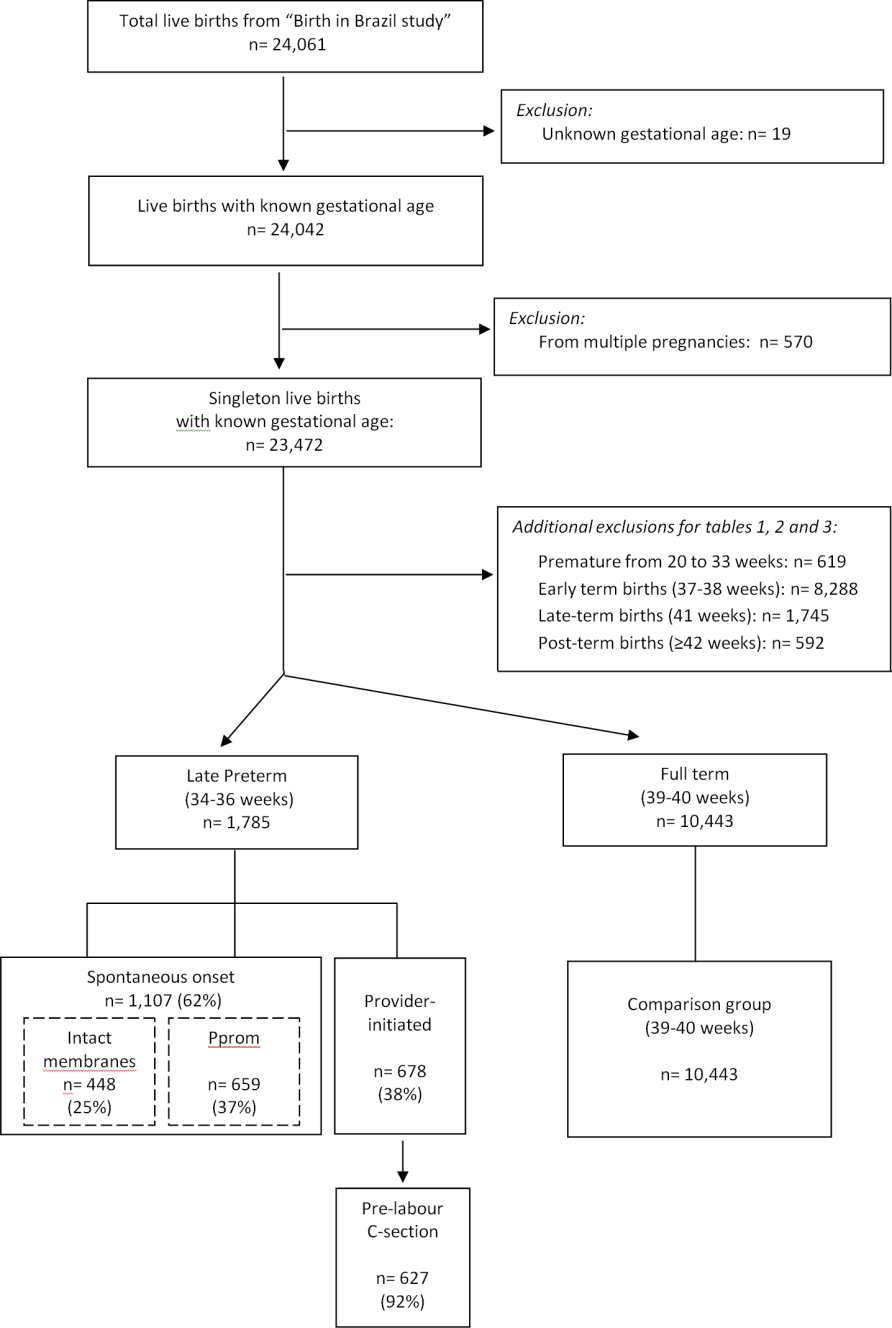
Flowchart.

Our final sample included 23,472 singleton live births with known GA. Of these, 10,443 were full-term, defined as GA at birth between 39 ^0/7^ and 40 ^6/7^ weeks of gestation and 1,785 were late preterm, defined as GA at birth between 34 ^0/7^ and 36 ^6/7^ weeks of gestation. A further 11,244 newborns were of other GA at birth. Gestational age was calculated using an algorithm that primarily relied upon early ultrasound estimates [18]. For analysis of provider-initiated-late-preterm birth associated factors we also excluded premature deliveries at 20–33 weeks gestation, term deliveries at 37, 38 or 41 weeks gestation and all post-term deliveries (≥42 weeks gestation) (Fig 1). Excluding these selected GA ranges ensured a comparison group with a lower prevalence of factors related to early or late GA.

### Outcome variable

The primary outcome was the incidence of provider-initiated late preterm births, i.e., those triggered by an obstetric intervention (induction of labour or pre-labour C-section), as opposed to spontaneous late preterm births, which either initiated spontaneously or by premature rupture of membranes (pProm).

Firstly, we analysed factors associated with provider-initiated late preterm births relative to full-term births, stratifying by public and private health services. Secondly, we analysed differences in the rate of provider-initiated late preterm births between public and private health services, stratifying by obstetric risk (described below). The rates were calculated by dividing the number of provider-initiated late preterm births by the number of singleton live births of all gestational ages.

### Exposure variables and definitions

We evaluated maternal age, years of schooling, parity, previous preterm birth(s), previous C-section, non-cephalic presentation, and obstetric risk characteristics, which were analysed separately and in combination. Women were classified to have high obstetric risk if they had maternal and/or newborn health problems, as well as obstetric complications. These included hypertensive disorders (chronic hypertension, preeclampsia, and HELLP syndrome); eclampsia; pre-existing and gestational diabetes; renal, cardiac or autoimmune diseases; severe infection at admission for birth; placental abruption; placental previa; intrauterine growth restriction (IUGR); or severe newborn malformations (those potentially related to indication for C-section and/or prematurity). Age and schooling were collected through interviewing the mother, and all other variables were extracted from medical records.

The obstetric risk was independently performed and validated by two obstetricians who also classified the prematurity determining factor (as spontaneous, Pprom or provider-initiated).

Women who gave birth in public or mixed-funding hospitals, but who were not covered by private health insurance plans, were classified as receiving public health services/care at childbirth. Women whose birth was covered by a private health insurance plan, and those who gave birth in private hospitals regardless of coverage by a health insurance plan, were classified as receiving private health services/care at childbirth.

### Statistical analysis

We analysed differences in participant characteristics between public and private health services using chi square. Differences in the distribution of the determining factor (spontaneous, Pprom or provider-initiated) between public and private health services were also analysed using chi square.

To assess provider-initiated late prematurity associated factors relative to full-term births, we performed non-conditional multiple logistic regressions stratified by source of healthcare provision (public or private). We report the estimated crude odds ratio (OR) and adjusted odds ratio (adjOR) with 95% confidence intervals (CI).

We analysed differences in the rate of provider-initiated late preterm births between public and private health services by means of non-conditional multiple logistic regressions, stratified by maternal obstetric risk (low or high). We report the estimated crude OR and adjOR with 95% CI. We performed two different adjustments for confounders. First, we adjusted for maternal age, years of schooling, parity, previous preterm birth(s), and non-cephalic presentation. Second, we adjusted for all these variables plus previous C-section.

In all statistical analyses, the complex sampling design was taken into consideration. A significance level of 5% was adopted for all analyses. The statistical program used was SPSS, version 20.0 (SPSS Inc., Chicago, IL, USA).

### Ethical approval

This study was carried out in accordance with the National Health Council Resolution n. 196/96. The ethics committee of the Sérgio Arouca National School of Public Health, Oswaldo Cruz Foundation (CEP/ENSP), approved this study under the research protocol CAAE: 0096.0.031.000–10 (approval date: May 11^th^ 2010). All hospital directors and postnatal women gave written informed consent.

## Results

Of the 1,785 late preterm births identified in this study, 1,107 (62%) were spontaneous: 448 (25%) with spontaneous onset of labour and 659 (37%) with Pprom. The remaining 678 (38%) were provider-initiated, among which 627 (92%) were by pre-labour C-section (Fig 1).

Maternal characteristics varied according to source of healthcare provision, in both late preterm and full-term births. Users of public health services were younger, had lower schooling levels, higher parity, and fewer previous C-sections. There were no differences in maternal and neonatal pathologies, or obstetric complications between the public and private health services, except for placental abruption, which was higher in public services. Among late preterms, 64% were born to mothers with no obstetric risk, without significant difference between type of health services received (Table 1).

**Table 1:** Characteristics of women by gestational age and type of childbirth care in singleton live births. Birth in Brazil, 2011–2012.

	Public		Private		P-value* Public vs. Private
	Late preterm (34–36 weeks)	Full term (39–40 weeks)	Late preterm (34–36 weeks)	Full term (39–40 weeks)	
	n (%)	n (%)	n (%)	n (%)	
**Total**	1,415 (100.0)	8,576 (100.0)	370 (100.0)	1,867 (100.0)	
**Age**					
12 to 19	382 (27.0)	1,845 (21.5)	18 (4.9)	106 (5.7)	<0.001
20 to 34	903 (63.8)	6,067 (70.7)	284 (76.8)	1,451 (77.7)	
≥ 35	130 (9.2)	664 (7.7)	68 (18.4)	310 (16.6)	
**Years of schooling**					
≤ 7	479 (33.9)	2,559 (29.8)	12 (3.2)	73 (3.9)	<0.001
8 to 10	440 (31.1)	2,649 (30.9)	41 (11.1)	181 (9.7)	
11 to 14	444 (31.4)	3,128 (36.5)	212 (57.3)	948 (50.8)	
≥ 15	52 (3.7)	240 (2.8)	105 (28.4)	665 (35.6)	
**Previous births**					
0	680 (48.1)	3,873 (45.2)	191 (51.6)	1,067 (57.2)	<0.001
1 to 2	556 (39.3)	3,760 (43.8)	164 (44.3)	767 (41.1)	
≥ 3	179 (12.7)	943 (11.0)	15 (4.1)	33 (1.8)	
**Previous preterm birth**^**1**^					
No	549 (74.6)	4,283 (91.1)	130 (72.6)	738 (92.3)	0.874
Yes	187 (25.4)	421 (9.0)	49 (27.4)	62 (7.8)	
**Previous caesarean section**^**1**^					
No	513 (69.7)	3,028 (64.4)	65 (36.3)	273 (34.1)	<0.001
1	149 (20.2)	1,232 (26.2)	91 (50.8)	456 (57.0)	
≥ 2	74 (10.1)	443 (9.4)	23 (12.8)	71 (8.9)	
**Newborn presentation**					
Cefalic	1,347 (95.1)	8,299 (96.7)	350 (94.6)	1,797 (96.2)	0.185
Non-cefalic	70 (4.9)	279 (3.3)	20 (5.4)	71 (3.8)	
**Obstetric risk**					
Hypertensive disorders^2^	265 (18.7)	765 (8.9)	91 (24.6)	136 (7.3)	0.822
Eclampsia	21 (1.5)	34 (0.4)	10 (2.7)	7 (0.4)	0.242
Preexisting Diabetes	33 (2.3)	82 (1.0)	4 (1.1)	14 (0.7)	0.144
Gestational Diabetes	157 (11.1)	707 (8.2)	47 (12.7)	128 (6.9)	0.207
Severe chronic diseases^3^	18 (1.3)	49 (0.6)	4 (1.1)	23 (1.2)	0.090
Infection at hospital admission for birth	10 (0.7)	20 (0.2)	4 (1.1)	2 (0.1)	0.860
Abruptio placentae	45 (3.2)	91 (1.1)	8 (2.2)	6 (0.3)	0.004
Placental praevia	13 (0.9)	33 (0.4)	10 (2.7)	4 (0.2)	0.311
IUGR	101 (7.1)	395 (4.6)	51 (13.8)	64 (3.4)	0.713
Major newborn malformation^4^	4 (0.3)	5 (0.1)	1 (0.3)	1 (0.1)	0.993
**Any of the above**	485 (34.3)	1,818 (21.2)	160 (43.2)	336 (18.0)	0.405
**Determining factor**					
Spontaneous	638 (45.1)	4,725 (55.1)	53 (14.3)	298 (16.0)	<0.001
Prom	290 (20.5)	873 (10.2)	91 (24.6)	120 (6.4)	
Provider initiated	489 (34.6)	2,980 (34.7)	226 (61.1)	1,450 (77.7)	

* χ2 test.

1 Considering women with at least one previous birth (public 34–36, n: 736; public 39–40, n:4704; private 34–36 n:179; private 39–40 n:800).

2 Chronic hypertension, pre-eclampsia and hellp syndrome.

3 Chronic renal diseases, chronic cardiac diseases and auto-imune diseases.

4 Malformations potentially related to caesarean section indication and/or prematurity.

Regarding late preterms in women receiving public healthcare, 28.6% of labours began spontaneously, 39.4% were preceded by Pprom, and 31.9% were provider-initiated. In women receiving private healthcare, these values were 11.6%, 27.3%, and 61.1%, respectively. For full-term infants, most births in women receiving public healthcare were spontaneous or preceded by Pprom, while in women receiving private healthcare 75.9% were provider-initiated (Table 2).

**Table 2:** Determining factor and type of delivery by gestational age and type of childbirth care in singleton live births. Birth in Brazil, 2011–2012.

	Public	Private	P-value*	Total
	n (%)	n (%)		n (%)
**34–36 weeks**				
**Total**	1,415 (100.0)	370 (100.0)		1,785 (100.0)
**Spontaneous**	405 (**28.6**)	43 (**11.6**)	<0.001	448 (**25.1**)
Vaginal	332	19		351
Caesarean	73 (18.0)	24 (55.8)		97 (21.7)
**Pprom**	558 (**39.4**)	101 (**27.3**)	0.001	659 (**36.9**)
Vaginal	415	22		437
Caesarean	143 (25.6)	79 (78.2)		222 (33.7)
Pre-labour CS	115 (80.2)	70 (88.5)		185 (83.4)
**Provider initiated**	452 (**31.9**)	226 (**61.1**)	<0.001	678 (**38.0**)
Vaginal	46	5		51
Caesarean	406 (89.8)	221 (97.8)		627 (92.5)
Pre-labour CS	404 (99.5)	221 (100.0)		625 (99.7)
**39–40 weeks**				
**Total**	8,576 (100.0)	1,867 (100.0)		10,443 (100.0)
**Spontaneous**	3,171 (**37.0**)	220 (**11.8**)	<0.001	3,391 (**32.5**)
Vaginal	2,639	153		2,792
Caesarean	532 (16.8)	67 (30.5)		599 (17.7)
**Prom**	2,576 (**30.0**)	230 (**12.3**)	<0.001	2,806 (**26.9**)
Vaginal	2,036	110		2,146
Caesarean	540 (21.0)	120 (52.2)		660 (23.5)
Pre-labour CS	426 (78.8)	95 (79.2)		521 (78.9)
**Provider initiated**	2,829 (**33.0**)	1,417 (**75.9**)	<0.001	4,246 (**40.7**)
Vaginal	398	22		420
Caesarean	2,431 (85.9)	1,395 (98.4)		3,826 (90.1)
Pre-labour CS	2,415 (99.3)	1,392 (99.8)		3,807 (99.5)

* χ2 test.

In public health services, 18.0% of women who went into late preterm spontaneous labour and 25.6% who had a Pprom underwent a C-section. In women receiving private care, C-section proportions were three-fold higher for these two groups. C-section procedures were mostly pre-labour, regardless of the determining factor (Pprom or provider-initiated), gestational age (late preterm or full term) or type of health services (public or private) (Table 2).

In the adjusted analysis, factors associated with provider-initiated late prematurity were the same in public and private health services, except for age above 35 and previous C-section, which were only associated with public healthcare provision. The strength of the association was higher for almost all pathologies in women receiving private healthcare (Table 3).

**Table 3:** Factors associated with provider-initiated late preterm birth (34–36 wks) relative to full term birth (39–40 wks) by type of childbirth care in singleton live births. Birth in Brazil, 2011–2012.

	Public (n = 452 provided-initiated late preterm vs. 8,576 full term)			Private (n = 226 provided-initiated late preterm vs. 1,867 full term)		
Exposure variables	OR	OR adj.^1^	CI	OR	OR adj.^1^	CI
**Age**						
12 to 19	1.03	1.07	(0.79–1.44)	0.90	1.03	(0.41–2.60)
20 to 34	1.00	1.00	-	1.00	1.00	-
≥ 35	1.76	1.69	(1.20–2.39)	1.25	1.20	(0.75–1.93)
**Years of schooling**						
≤ 7	0.59	0.59	(0.33–1.04)	0.42	0.37	(0.14–1.00)
8 to 10	0.59	0.63	(0.35–1.13)	1.03	1.03	(0.57–1.88)
11 to 14	0.66	0.70	(0.41–1.20)	1.07	1.07	(0.77–1.49)
≥ 15	1.00	1.00	-		1.00	-
**Previous births**						
0	1,12	1.12	(0.84–1.50)	0.82	0.81	(0.60–1.09)
1 to 2	1.00	1.00	-	1.00	1.00	-
≥ 3	1.38	1.33	(0.87–2.04)	1.77	1.51	(0.44–5.13)
**Previous preterm birth**						
No	1.00	1.00	-	1.00	1.00	-
Yes	4.09	3.74	(2.54–5.52)	4.40	4.53	(2.35–8.75)
**Previous caesarean section**						
No	1.00	1.00	-	1.00	1.00	-
Yes	1,78	1.64	(1.21–2.22)	1.13	1.04	(0.52–2.12)
**Newborn presentation**						
Cefalic	1.00	1.00	-	1.00	1.00	-
Non-cefalic	2,78	2.67	(1.76–4.06)	1.01	0.95	(0.52–1.73)
**Obstetric risk**						
Hypertensive disorders^2^	6,56	5.99	(4.33–8.29)	7.37	8.18	(5.68–11.8)
Eclampsia	10,16	9.54	(4.90–18.57)	11.51	11.82	(3.38–41.39)
Preexisting Diabetes	5,01	4.73	(2.38–9.43)	1.64	1.38	(0.31–6.26)
Gestational Diabetes	2,51	2.36	(1.69–3.29)	2.80	2.66	(1.73–4.08)
Severe chronic diseases^3^	3,98	3.66	(1.06–12.64)	1.62	1.59	(0.59–4.32)
Infection at hospital admission for birth	2,92	1.78	(0.53–6.02)	14.60	12.74	(2.33–69.55)
Placental abruption	6,11	5.40	(3.01–9.75)	10.40	9.25	(3.09–27.67)
Placental praevia	4,85	3.55	(1.08–11.68)	16.73	15.02	(4.63–48.81)
IUGR	3.88	3.83	(2.51–5.82)	6.95	6.73	(2.96–15.28)
Severe newborn malformation^4^	9,76	5.08	(0.64–39.98)	7.33	7.67	(0.55–106.42)
Any of the above (high obstetric risk)	6,63	6.34	(4.63–8.69)	7.00	7.10	(5.32–9.48)

1 Adjusted for age, years of schooling, parity, previous preterm bith, previous CS, non-cefalic presentation and obstetric risk.

2 Chronic hypertension, pre-eclampsia and hellp syndrome.

3 Chronic renal diseases, chronic cardiac diseases and auto-imune diseases.

4 Malformations potentially related to caesarean section indication and/or prematurity.

The rate of provider-initiated late preterm birth was of 2.9%–2.4% for women receiving public health services and 4.8% for women receiving private health services. After adjusting for confounders, women receiving private healthcare had higher odds of provider-initiated late preterm birth regardless of obstetric risk. For women of low obstetric risk the adjusted OR was of 2.3 (CI 1.5–3.6) and for the ones of high obstetric risk the adjusted OR was of 1.6 (CI 1.1–2.3) (Table 4).

**Table 4:** Private childbirth care associated with provider-initiated late preterm rate (%) in singleton live births. Birth in Brazil, 2011–2012.

	Singleton live births	Provider-initiated late preterm birth		Absolute rate diferences	Crude OR	adj. OR^1^ (CI)		adj. OR^2^ (CI)	
	n	n	Rate (%)						
**All women**	23,472	678	2.9						
Public	18,809	452	2.4						
Private	4,663	226	4.8	2.4	2.07	1.82	(1.27–2.40)	1.65	(1.21–2.25)
**Low obstetric risk women**	17,945	248	1.4						
Public	14,443	159	1.1						
Private	3,502	89	2.5	1.4	2.35	2.34	(1.53–3.58)	2.15	(1.41–3.30)
**High obstetric risk women**	5,527	430	7.8						
Public	4,366	293	6.7						
Private	1,161	137	11.8	5.1	1.86	1.60	(1.10–2.32)	1.58	(1.09–2.27)

1 Adjusted for age, years of schooling, parity, previous preterm bith and non-cefalic presentation.

2 Adjusted for age, years of schooling, parity, previous preterm bith, non-cefalic presentation and previous CS.

## Discussion

### Main Findings

In Brazil, one in nine births is preterm (S2 Table) [21]. In this study, late prematurity accounted for 75% of all preterm births, and around 40% of these were provider-initiated births. Labour induction practice was low in public health services, and almost nil in private health services. Provider-initiated late prematurity was associated with previous preterm birth(s) and maternal pathologies in both health services, and with maternal age ≥35 years in public health services only. The higher rate of provider-initiated late preterm birth in women receiving private healthcare compared with the ones receiving public care was independent of obstetric risk.

### Strengths and Limitations

The strength of this study is that we have used a representative nationwide survey, with primary data collected from medical records. This allowed, for the first time, a description of the national late prematurity rate and its determinants, as well as a more accurate gestational age estimate calculated by an algorithm that primarily relied upon early ultrasound estimates [18]. Furthermore, we stratified the analysis by source of healthcare provision (public or private) and according to obstetric risk (low or high). The obstetric risk was independently performed and validated by two obstetricians who also classified the prematurity determining factor (as spontaneous, Pprom or provider-initiated). Nonetheless, we failed to analyse whether C-section indication was appropriate on a case-by-case basis.

### Interpretation

Maternal age, previous C-section, and non-cephalic presentation were not associated with provider-initiated late prematurity in private health services, as in the public services. This could be attributed to the indiscriminate use of interventions in private health services, independent of a patient’s characteristics, as well as the high prevalence of the interventions in the full-term comparison group. Nakamura-Pereira et al. (2016) have previously observed that in private health services C-section rate in cephalic presentation preterms was 77%, even among women at low obstetric risk (S1 Table) [10].

Women receiving public healthcare live in poorer socioeconomic conditions and face barriers of access to prenatal care more frequently [24, 25]. However, the prevalence of maternal morbidities was comparable to women receiving private healthcare. On the other hand, we observed a stronger association between these morbidities and provider-initiated late prematurity in private health services. This may indicate greater anticipation of birth even under the same health conditions. Possible explanations would be the easier access to neonatal ICU in private hospitals, as well as unawareness or minimization of the risks of late preterm infants, who are generally still immature [22].

In the short term, late preterm infants are vulnerable to thermal instability, breastfeeding difficulties, hypoglicemia, hyperbilirubinemia, infections, and respiratory morbidities. This leads to a greater need for neonatal ICU and readmission after birth [26, 27]. In the long term, they have a higher risk of respiratory diseases, infant hospitalisation, neurodevelopmental problems, and poorer school performance [26–28]. Probably many infants in Brazil are unnecessarily being placed at risk for these outcomes.

Our data reaffirm that the prevalence of provider-initiated births in Brazil is one of the highest worldwide. This is especially true in private health services, which shows an inverted pattern of two thirds of births determined by an obstetric intervention. In contrast, two thirds of preterm births from high-income countries occur spontaneously [6, 15]. The major intervention characterising Brazilian obstetric care is C-section. We observed that 53% of late preterm births were by C-section, among which 86% were pre-labour C-section. Unsurprisingly, labour induction practice corresponded to less than 10% of births; around 20% in public health services and only 4% in the private.

Brazilian private health services schedule birth care to optimise physician’s time and usually the same physician provides both prenatal and birth care. As such, several deliveries are set for the same date, in agreement with the pregnant women [8, 29, 30]. Thus, receiving prenatal and birth care from the same practitioner turned out to be the highest risk factor for having a C-section [22]. This is probably the main reason rates of pre-labour C-section are particularly high in Brazilian private health services. [7, 10, 22, 29].

C-section can impact negatively woman's obstetric life. There is substantial evidence that C-section increases the risk of placenta previa and accreta in subsequent pregnancies [31], severe acute maternal morbidity [32], and mortality [33]. In addition, newborns endure losses in breastfeeding [12] and have smaller diversification of the intestinal microbiota [34], which has been related to chronic diseases in adult life [35–38].

We found higher odds of provider-initiated late preterm birth in women receiving private care compared with the ones receiving public healthcare. This was regardless of the obstetric risk classification of the women (low or high) and adjustment for confounding variables. However, when adjusted for previous C-section, there was a reduction in the OR for low-risk women. This implies that, for low-risk women, the association was partially explained by a higher prevalence of previous C-section in private health services. This was not evident in the adjusted model of Table 3 owing to the extremely high frequency of mothers with previous C-section in the full-term comparison group from private health services.

According to recommendations from the American Congress of Obstetricians and Gynecologists [39], most women with one previous C-section with a low-transverse incision are candidates for and should be counselled about vaginal birth after cesarean (VBAC) and offered a trial of labour after C-section [40]. Studies have referred to a 70% VBAC success rate [41], which should be incorporated into Brazilian obstetric practice, as repetitive C-section further increases the risk of haemorrhage, abnormal placenta [31], and uterine rupture [42] in subsequent pregnancies.

In the USA, it has been quantified that one in five late preterm births were not registered as indicated for an intervention in the medical records [6]. Therefore, it was suggested that other non-clinical factors might have influenced the decision at the moment of intervention. The authors of the study strongly recommended the reduction of iatrogenic interventions, and suggested the use of guidelines to optimise and plan the timing and route of delivery. The Institute of Medicine (IOM) estimated a burden of prematurity in the USA of about $26 billion or $51,600 per child [43]. It is worth noting that provider-initiated prematurity has been reduced recently in the USA [44].

Excessive perinatal interventions (in mothers and newborns), independent of obstetric/fetal risks, have previously been reported in private health services of Brazil [9, 12] and Australia [45]. Our study estimated the effect on rates of prematurity if, among low-risk women, rates of provider-initiated late preterm births were the same in private services as in public services. We found that the prematurity rate would decrease to 11% (from 9.5% to 8.4%) in private health services. This would indicate an annual reduction of approximately 6,600 preterm births nationally. It is essential to highlight that there would be a higher impact on preterm birth rates if provider-initiated late preterm births were prevented in women of high obstetric risk as well. Although this group corresponded to 24% of women, provider-initiated late preterm birth rate was more than five times higher when compared to women of low obstetric risk; with additional differences between public and private services. Moreover, the potential for reduction of preterm birth rates in Brazil can be of greater magnitude if the comparison group is external (from high-income European countries) rather than Brazilian public health services, which is yet quite interventionist.

## Conclusion

We found a very high rate of provider-initiated late preterm births in Brazil, mostly performed by pre-labour C-section. It has been demonstrated that many of the indications for interventions to anticipate birth were not based on scientific evidence. This suggests a considerable potential for reduction as such prematurity can be avoided, as opposed to cases of spontaneous occurrence with an unknown cause. Excessive use of C-section will likely lead directly to increased costs for the healthcare system due to surgery, greater use of neonatal ICU, and maternal near misses. A further indirect cost is the association with short- and long-term morbidity of women and children. To reduce iatrogenic prematurity and promote healthy births, it is advisable to introduce policies with incentives for the adoption of new models of birth care in Brazil. In particular, these should be directed to adequate prenatal care for women with obstetric pathologies and appropriate indication of the timing of birth.

## Supporting Information

S1 File Database. (ZIP)

S1 Table Proportion of caesarean section by source of payment of childbirth and obstetric risk. Birth in Brazil study, 2011–2012.
(DOCX)

S2 Table Preterm delivery according to gestational age and determining factor. Birth in Brazil study, 2011–2012.
(DOCX)
