# Supplementary material for: Provider-Initiated Late Preterm Births in Brazil: Differences between Public and Private Health Services
Source: PLoS One. 2016 May 19;11(5):e0155511. doi: 10.1371/journal.pone.0155511 (PMC4873204; doi:10.1371/journal.pone.0155511)
Supplement: S1 Table — Birth in Brazil study, 2011–2012. (DOCX) [file pone.0155511.s002.docx]

| **S1 Table - Proportion of caesarean section by source of payment of childbirth and obstetric risk. Birth in Brazil study, 2011-2012** | | | | | | | |
| --- | --- | --- | --- | --- | --- | --- | --- |
|  |  | Public | | | Private | | |
| Robson group |  | LRW | HRW^1^ | *P-value^2^* | LRW | HRW^1^ | *P-value^2^* |
| 1 | Nulliparous, single cephalic, >=37 weeks, in spontaneous labour | 13.7 | 42.9 | <0,001 | 43.4 | 48.7 | 0.65 |
| 2 | Nulliparous, single cephalic, >=37 weeks, induced or CS before labour | 69.3 | 88.4 | <0,001 | 96.6 | 99.0 | 0.062 |
| 3 | Multiparous (excluding prev. CS), single cephalic, >=37 weeks, in spontaneous labour | 3.5 | 15.3 | <0,001 | 10.7 | 16.1 | 0.262 |
| 4 | Multiparous without a previous uterine scar, with single cephalic pregnancy, >=37 weeks, induced or CS labour | 47.6 | 69.3 | <0,001 | 87.3 | 90.4 | 0.385 |
| 5 | Previous CS, single cephalic, >=37 weeks | 74.7 | 87.3 | <0,001 | 97.6 | 99.6 | 0.031 |
| 8 | All multiple pregnancies (including prev. CS) | 75.7 | 88.6 | 0.032 | 92.3 | 100.0 | 0.502 |
| **10** | **All single cephalic, <=36 weeks (including prev. CS)** | **25.4** | **71.4** | **<0,001** | **77.6** | **94.9** | **<0,001** |
| 6, 7 and 9 | All nulliparous breeches; All multiparous breeches (including prev. CS); All abnormal lies (including prev. CS) | 89.1 | 90.3 | 0.787 | 99.4 | 98.7 | 0.551 |
| LRW: low obstetric risk women; HRW: High obstetric risk women. | | | | | | | |
| 1 High obstetric risk covered the following complications: hypertensive disorders, eclampsia, preexisting diabetes, gestational diabetes, severe chronic diseases, severe infection during pregnancy, placental abruption, placenta praevia, intrauterine growth restriction and major newborn malformation. | | | | | | | |
| ^2^ χ2 test of differences in proportions of caesarean section between Low and High obstetric risk women. | | | | | | | |
