# Supplementary material for: Provider-Initiated Late Preterm Births in Brazil: Differences between Public and Private Health Services
Source: PLoS One. 2016 May 19;11(5):e0155511. doi: 10.1371/journal.pone.0155511 (PMC4873204; doi:10.1371/journal.pone.0155511)
Supplement: S2 Table — Birth in Brazil study, 2011–2012. (DOCX) [file pone.0155511.s003.docx]

| **S2 Table - Preterm delivery according to gestational age and determining factor. Birth in Brazil study, 2011-2012** | | | | |
| --- | --- | --- | --- | --- |
|  | **n** | **%** | ***rate*** | **95% CI** |
| **Total** | 2,771 | 100.0 | 11.5 | (10,3 - 12,9) |
| **Gestational age (weeks)*** |  |  |  |  |
| < 28 | 100 | 3.6 | 0.4 | (0,3 - 0,6) |
| 28-31 | 333 | 12.0 | 1.4 | (1,0 - 1,9) |
| 32-33 | 288 | 10.4 | 1.2 | (0,9 - 1,5) |
| 34-36 | 2,050 | 74.0 | 8.5 | (7,7 - 9,4) |
| **Determining factor*** |  |  |  |  |
| Spontaneous | 1,681 | 60.7 | 7.0 | (6,2 - 7,9) |
| Intact membranes | 660 | 23.8 | 2.7 | (2,3 - 3,2) |
| Pprom | 1,021 | 36.9 | 4.2 | (3,8 - 4,7) |
| Provider-initiated | 1,090 | 39.3 | 4.5 | (4,1 - 5,1) |
| Labor induction | 60 | 2.2 | 0.2 | (0,1 - 0,8) |
| Prelabor Cesarean | 1,030 | 37.2 | 4.3 | (4,0- 4,7) |
| * For each category or sub-category the rate was calculated by dividing the number of newborns by the total number of live births, of 24,042. | | | | |
